# Combined effects of nitrate and medium-chain fatty acids on methane production, rumen fermentation, and rumen bacterial populations in vitro

**DOI:** 10.1038/s41598-023-49138-6

**Published:** 2023-12-11

**Authors:** Mariana Vadroňová, Adam Šťovíček, Kateřina Jochová, Alena Výborná, Yvona Tyrolová, Denisa Tichá, Petr Homolka, Miroslav Joch

**Affiliations:** 1 Department of Microbiology, Nutrition and Dietetics, Faculty of Agrobiology, Food and Natural Resources, Czech University of Life Sciences, Kamýcká 129, 165 00 Prague, Czech Republic; 2 Department of Nutrition and Feeding of Farm Animals, Institute of Animal Science, Přátelství 815, 104 00 Prague, Czech Republic

**Keywords:** Microbiome, Climate-change mitigation, Animal physiology

## Abstract

This study investigated the combined effects of nitrate (NT) and medium-chain fatty acids (MCFA), including C8, C10, C12, and C14, on methane (CH_4_) production, rumen fermentation characteristics, and rumen bacteria using a 24 h batch incubation technique. Four types of treatments were used: control (no nitrate, no MCFA), NT (nitrate at 3.65 mM), NT + MCFA (nitrate at 3.65 mM + one of the four MCFA at 500 mg/L), and NT + MCFA/MCFA (nitrate at 3.65 mM + a binary combination of MCFA at 250 and 250 mg/L). All treatments decreased (*P* < 0.001) methanogenesis (mL/g dry matter incubated) compared with the control, but their efficiency was dependent on the MCFA type. The most efficient CH_4_ inhibitor was the NT + C10 treatment (− 40%). The combinations containing C10 and C12 had the greatest effect on bacterial alpha and beta diversity and relative microbial abundance (*P* < 0.001). Next-generation sequencing showed that the family *Succinivibrionaceae* was favored in treatments with the greatest CH_4_ inhibition at the expense of *Prevotella* and *Ruminococcaceae*. Furthermore, the relative abundance of *Archaea* decreased (*P* < 0.05) in the NT + C10 and NT + C10/C12 treatments. These results confirm that the combination of NT with MCFA (C10 and C12 in particular) may effectively reduce CH_4_ production.

## Introduction

Globally, the sustainability of agricultural systems relies on efficient livestock production^1^. Naturally, ruminants lack total nutritional efficiency, as they waste 2–15% of their ingested energy^2^ by emitting enteric methane (CH_4_)^3^. CH_4_ is now recognized as a major global concern and one of the causes of climate change^4^. Various feed additives have been explored to effectively mitigate CH_4_ emissions^5^, including 3-nitrooxypropanol^3^ and red seaweed (*Asparagopsis* spp.)^6^. However, rumen CH_4_ is yet to be effectively mitigated in a cost-effective and practical manner for adoption at the farm level^5,7,8^. Farmers are more likely to adopt a CH_4_ mitigating option that is particularly economical as well as nutritionally and environmentally beneficial^5^.

One possible solution for countering fermentation inefficiency is to combine anti-methanogenic inhibitors with complementary modes of action. For example, nitrate (NT) and dietary fat decrease CH_4_ additively^9–11^. The effects of NT have been proven^10^, but the efficacy of lipids depends on their form, concentration, and fatty acid composition^12,13^. One of the most effective types of lipids for CH_4_ mitigation are medium-chain fatty acids (MCFA) and polyunsaturated fatty acids (PUFA)^13^, with MCFA being the most effective^14–16^. MCFA include caprylic (C8), capric (10), lauric (C12), and myristic acids (C14)^16^. MCFA have been supplemented in pure forms or in products rich in them (e.g., oils)^17^; however, it is unclear which MCFA inhibit methanogenesis^18^. Pure forms of MCFA, as well as their various sources and combinations, affect the rumen differently^17,19^. For example, C12 and C14 have been extensively evaluated; in addition to being effective individually, their combination has a synergistic effect in reducing CH_4_^15,20^.

NT and MCFA are effective CH_4_ inhibitors when used alone; however, their high concentrations have adverse effects on rumen fermentation and feed intake^10^. Caution must be taken when supplementing NT because of the risk of nitrite poisoning^10^ and loss of high levels of nitrogen in urine^21^. Furthermore, high amounts of dietary lipids (8–9%), including MCFA, reduce dry matter intake (DMI), digestibility^22,23^, and consequently, production efficiency^24,25^.

NT mainly functions as an electron sink because NT reduction is thermodynamically more favorable than CO_2_ reduction^10^. Additionally, NT is toxic to protozoa^10^, and its intermediate nitrite is toxic to methanogens^26,27^. MCFA are antibacterial and antiprotozoal agents^19,28,29^ as they dissociate in bacterial cells^17^, may cause defaunation^30,31^, and, therefore, lower H_2_ supply^5,9^. Additionally, MCFA can directly inhibit methanogens^29,32^. The target microorganisms of NT and MCFA appear to overlap, but their effects may be complementary. Mixing low doses of NT and MCFA (and their binary combinations) may decrease rumen methane production without inhibiting fermentation.

Our hypotheses were as follows: (1) different combinations of MCFA and NT would vary in their effectiveness in mitigating CH_4_ production, and (2) efficient combinations of MCFA and NT capable of lowering methanogenesis without negatively affecting rumen fermentation may be found.

## Results

### Fermentation parameters

The results of the treatment effects on in vitro total gas (TGP) and CH_4_ production, apparent dry matter disappearance (aDMd), ammonia-N (NH_3_-N) concentration, and pH are summarized in Table 1. All treatment combinations of NT + MCFA significantly reduced CH_4_ production per dry matter incubated (DMi) for 24 h. The extent of reduction varied among the ten combinations tested. Compared with the unsupplemented control, the addition of NT + C10 and NT + C10/C12 led to the highest CH_4_ reduction, − 40% and − 34%, respectively (Table 1). A significant reduction (*P* < 0.001) with these two combinations was achieved without any negative effects on fermentation (assessed by net VFA (nVFA) produced) or substrate digestibility (assessed by aDMd; *P* > 0.05). nVFA production was affected by treatment (*P* = 0.031); however, specific multiple comparisons after correction using Dunnett’s test did not identify a significant difference between any treatments. The treatment effect on CH_4_ mitigation (mL/g DMi) then decreased as follows: NT + C8/C10 (− 29.6%) > NT + C10/C14 (− 28.5%) > NT + C8/C12 (− 26.3%) > NT + C8 (− 24.1%) > NT + C12 and NT + C12/C14 (− 23.6%) > NT (− 17.3%) > NT + C14 (− 15.9%). Similarly, other CH_4_ production parameters, that is, CH_4_ per percentage of TGP, aDMd, or VFA, significantly decreased (*P* < 0.001) in a similar manner, with the NT + C10 and NT + C10/C12 treatments being the most effective. NT + C14 was the only combination that reduced (*P* < 0.05) aDMd (by 6.7%) and the only combination that did not reduce (*P* < 0.05) TGP.

**Table 1:** Effects of MCFA + NT on in vitro gas and methane production, apparent dry matter disappearance (aDMd), ammonia-N concentration, and pH.

Treatment	TGP^†^ (mL/g DMi^§^)	Methane				aDMd^‡^ (g/g)	Net NH_3_–N (mg/100 mL)	pH
		(mL/g DMi)	(% TGP)	(mL/g aDMd)	(mol/mol nVFA^¶^)			
Control	298.7	36.5	12.22	57.6	0.2125	0.6345	13.1	5.90
NT	289.3*	30.2*	10.42*	48.1*	0.1746	0.6313	20.7*	6.06*
NT + C8	286.6*	27.7*	9.64*	42.4*	0.1599*	0.6539	20.9*	6.06*
NT + C10	263.8*	21.9*	8.29*	34.6*	0.1498*	0.6372	24.7*	6.27*
NT + C12	282.8*	27.9*	9.84*	43.7*	0.1592*	0.6405	21.0*	6.13*
NT + C14	291.7	30.7*	10.51*	51.9*	0.1727	0.5923*	21.3*	6.08*
NT + C8/C10	277.9*	25.7*	9.23*	39.3*	0.1825	0.6569	24.0*	6.18*
NT + C8/C12	284.0*	26.9*	9.48*	41.2*	0.1678*	0.6565	20.9*	6.13*
NT + C8/C14	288.8*	29.1*	10.06*	45.6*	0.1637*	0.6402	22.4*	6.05*
NT + C10/C12	271.0*	24.0*	8.88*	38.4*	0.1478*	0.6315	22.7*	6.20*
NT + C10/C14	278.8*	26.1*	9.36*	41.0*	0.1511*	0.6383	21.2*	6.15*
NT + C12/C14	283.1*	27.9*	9.85*	45.2*	0.1548*	0.6184	21.9*	6.09*
SEM	2.53	0.65	0.163	1.22	0.00484	0.00517	0.93	0.020
*P*-value	< 0.001	< 0.001	< 0.001	< 0.001	0.009	0.002	< 0.001	< 0.001

^†^Total gas production; ^‡^apparent dry matter disappearance; ^§^dry matter incubated; ¶net production of volatile fatty acids.

*Means within a column differ significantly (*P* < 0.05) from the corresponding control (0 mg/L). SEM, standard error of the mean.

The nVFA (*P* = 0.031) and the acetate: propionate ratio (*P* = 0.065) were not altered by any treatment. The effects of NT + MCFAs on VFA production and individual proportions are shown in Table 2. NT + C10 significantly decreased (*P* < 0.05) the molar proportion of acetate and increased (*P* < 0.05) the molar proportions of butyrate, valerate, and *iso*-valerate. Furthermore, butyrate proportions were significantly reduced (*P* < 0.05) with the following treatments: NT, NT + C14, and NT + C12/C14. Finally, only the NT + C8 and NT + C10/C12 treatments substantially decreased (*P* < 0.05) the molar proportion of valerate compared to the control.

**Table 2:** Impacts of MCFA + NT on in vitro volatile fatty acids production and proportion.

Treatment	nVFA^†^ (mmol/L)	Molar proportion of VFA (mol/100 mol)						A:P^‡^
		Acetate	Propionate	Butyrate	*iso*-butyrate	Valerate	*iso*-valerate	
Control	67.4	60.5	20.7	10.9	0.8	4.9	2.2	3.0
NT	67.9	62.9	20.0	9.5*	0.9	4.5	2.2	3.2
NT + C8	68.1	61.1	21.6	10.3	0.9	4.0*	2.1	2.9
NT + C10	57.8	54.9*	20.1	14.4*	1.3	6.7*	2.6*	2.8
NT + C12	68.5	61.2	20.3	10.0	1.3	5.1	2.2	3.1
NT + C14	71.1	62.1	20.3	9.5*	1.1	4.7	2.3	3.1
NT + C8/C10	60.0	60.1	20.0	12.0	0.4	5.2	2.3	3.1
NT + C8/C12	65.4	60.1	21.4	10.6	0.7	4.9	2.2	2.8
NT + C8/C14	70.1	60.5	21.5	9.9	1.7	4.3	2.2	2.8
NT + C10/C12	64.0	57.3	21.8	11.6	1.2	5.8*	2.3	2.7
NT + C10/C14	67.9	59.4	21.4	10.5	1.5	4.9	2.1	2.8
NT + C12/C14	70.9	60.7	21.3	9.5*	1.5	4.9	2.1	2.8
SEM	1.65	0.40	0.33	0.31	0.12	0.19	0.08	0.05
*P*-value	0.031	< 0.001	0.067	< 0.001	0.198	< 0.001	0.013	0.065

^†^Net production of volatile fatty acids; ^‡^acetate:propionate.

*Means within a column differ significantly (*P* < 0.05) from the corresponding control (0 mg/L). SEM, standard error of the mean.

The pH increased (*P* < 0.05) in all treatments relative to the control; the highest increase was recorded for the NT + C10. NH_3_–N concentrations increased (*P* < 0.05) in all NT + MCFA treatments, with NT + C10 increasing NH_3_–N concentrations the most.

### Microbial community

**Figure 1:**
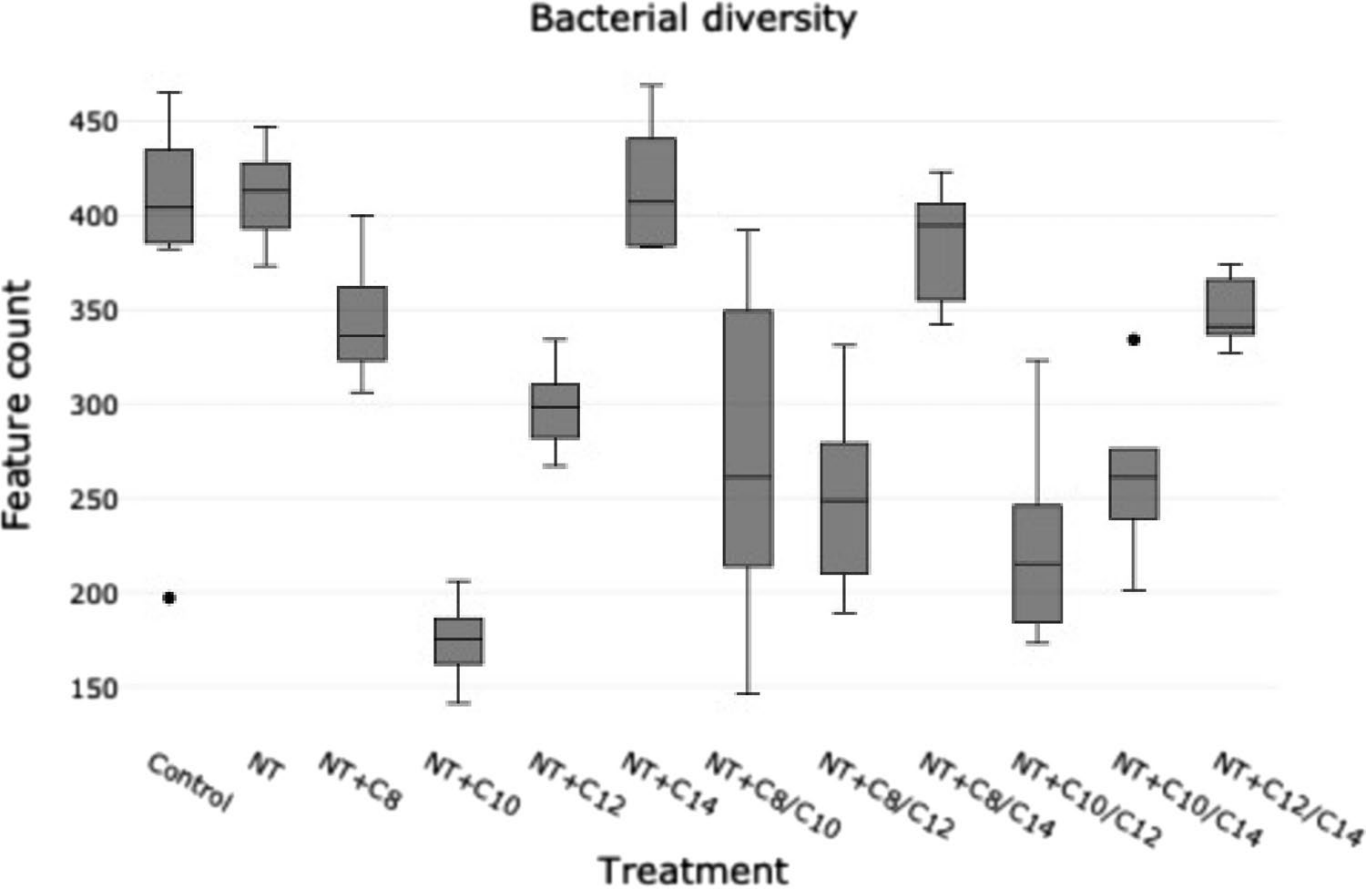
Stack-graphs showing the effect of NT + MCFA on bacterial diversity.

Bacterial diversity varied greatly among treatments and was expressed as ASV counts (Fig. 1) and indices of diversity (Table 3). All indices of alpha diversity significantly (*P* < 0.05) decreased when the treatments contained C10 or C12, with the NT + C10 treatment having the strongest effect on microbial diversity, richness, and evenness (Figs. 1, 4, Table 3). In contrast, the smallest effect on diversity was observed for treatments containing C8 and C14. Additionally, the NT + C10 and NT + C10/C12 treatments significantly decreased (*P* < 0.05) the relative abundance of Archaea (Table 4).

**Table 3:** The influence of the NT + MCFA treatments on α-diversity of microbial communities in vitro.

Treatment	Shannon diversity index	Simpson diversity index	Chao1 richness index	Pielou evenness index
Control	6.954	0.955	584	0.758
NT	7.101	0.965	588	0.773
NT + C8	5.856	0.878	507	0.653
NT + C10	3.171*	0.566*	259*	0.396*
NT + C12	5.125*	0.802	444*	0.584*
NT + C14	6.912	0.949	594	0.752
NT + C8/C10	4.922*	0.762*	437*	0.560*
NT + C8/C12	4.271*	0.703*	382*	0.499*
NT + C8/C14	6.504	0.925	573	0.712
NT + C10/C12	3.701*	0.622*	336*	0.441*
NT + C10/C14	4.524*	0.731*	390*	0.526*
NT + C12/C14	5.717	0.856	520	0.635
SEM	0.2287	0.0238	19.2	0.0220
*P*-value	< 0.0001	< 0.0001	< 0.0001	< 0.0001

*Means within a column differ significantly (*P* < 0.05) from the corresponding control (0 mg/L). SEM, standard error of the mean.

**Table 4:** Effects of MCFA + NT on the relative abundances of microbial communities at the kingdom level in vitro.

Kingdom Treatment	Bacteria	Archaea
Control	97.66	2.34
NT	98.57	1.43
NT + C8	98.58	1.42
NT + C10	99.63*	0.37*
NT + C12	98.60	1.40
NT + C14	98.48	1.52
NT + C8/C10	98.70	1.30
NT + C8/C12	98.85	1.15
NT + C8/C14	97.58	2.42
NT + C10/C12	99.10*	0.90*
NT + C10/C14	98.79	1.21
NT + C12/C14	98.78	1.22
SEM	0.005	0.003
*P*-value	0.023	0.023

*Means within a column differ significantly (*P* < 0.05) from the corresponding control (0 mg/L). SEM, standard error of the mean.

**Figure 2:**
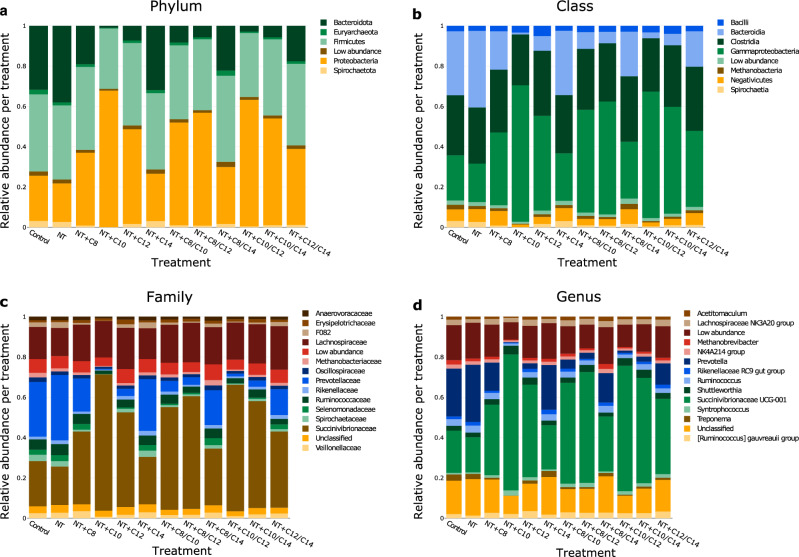
Bar-charts displaying the different effects of NT + MCFA on rumen microbiota at the phylum (**a**), class (**b**), family (**c**), and genus (**d**) levels.

The most abundant phyla (Fig. 2a) were *Proteobacteria* (relative abundance range 19.5–68.4%), followed by *Firmicutes* (30.2–43.8%), and *Bacteroidota* (0.96–37.98%). The dominance of the phylum *Proteobacteria* was not consistent across all treatments, and the dominance of the respective phyla varied based on the type of MCFA. *Proteobacteria* thrived in the C10 treatment group at the expense of *Bacteroidota*. The phylum *Bacteroidota* showed signs of particular sensitivity to MCFA, as its relative abundance was highest in the NT treatment. *Firmicutes*, conversely, appear to be favored in treatments containing C8.

To gain further insight into the bacterial community, the relative microbial abundances are also shown at the class (Fig. 2b), family (Fig. 2c), and genus (Fig. 2d) levels. The highest relative abundance at the family level was noted for *Succinivibrionaceae* (25.2–70.7%), followed by *Prevotellaceae* (0.5–34.5%) and *Lachnospiraceae* (14.9–23.2%). In general, the family *Succinivibrionaceae* showed an increasing trend in treatments containing C10 and/or C12. In contrast, the C10 treatment simultaneously decreased the relative abundance of *Prevotellaceae*.

**Figure 3:**
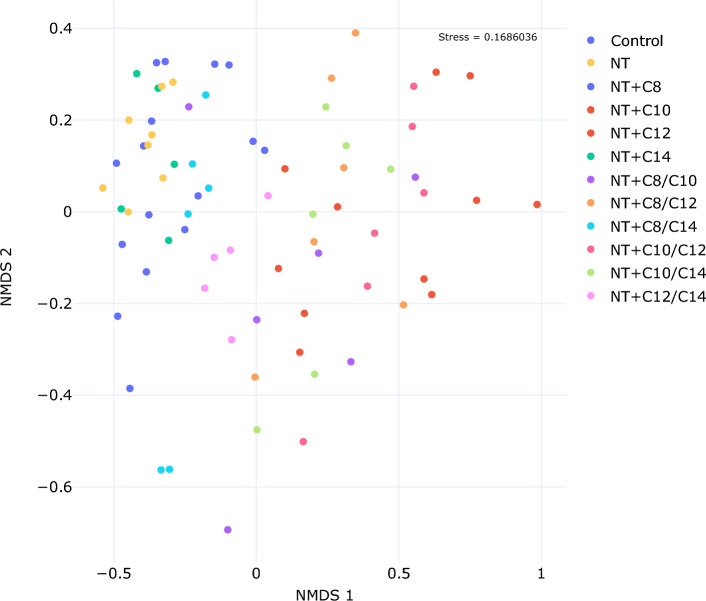
A nonmetric multidimensional scaling (NMDS) visualization depicting the microbiome variations at the ASV level in in vitro cultures based on the treatment applied (stress = 0.1686036). The plot clearly illustrates the impact of different treatments on microbiome composition. The treatments involving control, NT, C8, and C14 form a cluster in the upper-left part of the plot, whereas treatments with C10 and C12 are more situated towards the right side.

**Figure 4:**
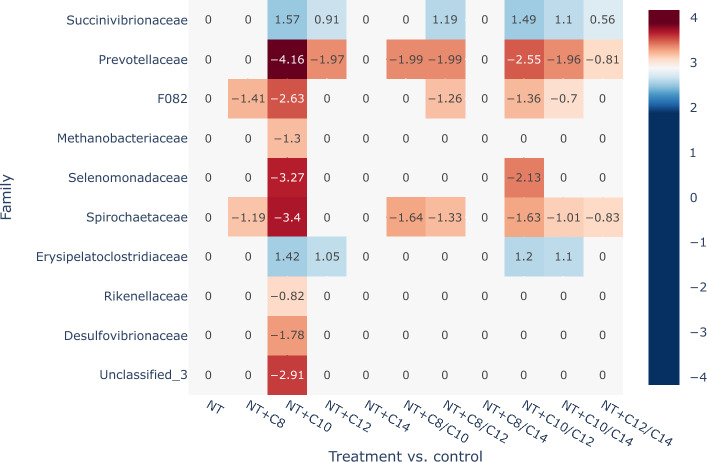
Analysis of Compositions of Microbiomes with Bias Correction (ANCOM-BC) shows positive log fold change (enriched taxa) and negative log fold change (decreased taxa) compared to control. Nonsignificant results (*P* > 0.05) were replaced by 0. At the family level, the addition of C10 had the most prominent effect on microbiota.

Overall, compared with the control, the treatments containing C10 and C12 had the strongest effect on the relative abundances of bacteria and Archaea. The weakest antimicrobial activity was observed in the C8 and C14 treatments and their respective combinations. Compared to the combination with MCFA, NT alone had little effect on the composition of the microbiota in this experiment. This was confirmed by analysis of Compositions of Microbiomes with Bias Correction (ANCOM-BC) implemented at a family level (Fig. 4) and nonmetric multidimensional scaling (NMDS) analysis used to visualize and assess community composition (Fig. 3).

In the NMDS analysis, the treatments exhibited distinct clustering patterns on the plot. Specifically, treatments comprising control, NT, C8, and C14 formed a cluster located in the upper-left quadrant of the plot (Fig. 3), indicating a high degree of similarity in their microbiome compositions. Conversely, treatments involving C10 and C12 displayed a tendency to be positioned towards the right side of the plot, suggesting differences in microbiome composition compared to the former group.

## Discussion

By combining low doses of NT and MCFA, we confirmed our hypotheses that (1) individual combinations varied in their CH_4_ mitigating efficacy and (2) there was one superior treatment (NT + C10) that decreased methanogenesis the most without negative effects on fermentation. In addition, the microbial community shifted to resemble the rumen microbiota of low-CH_4_ emitting cattle, with low *Ruminococcaceae*^33,34^ (e.g., 4.3%^33^), and high *Succinivibrionaceae*^35^ (e.g., 8.9–9.3%^36,37^).

Combinations of NT and various MCFA differed in their CH_4_ suppressing effect, confirming previous findings that CH_4_ mitigation is dependent on the MCFA type^19^. Overall, our results suggested that the most effective combinations were those containing C10. The most effective treatment was NT + C10 (− 40%; mL/g DMi). This is consistent with the anti-methanogenic effect of C10 reported by Goel et al. (2009), who showed a dose-dependent effect of C10^17^. In their study, C10 at 400 and 600 mg/L of incubation medium decreased CH_4_ production by 44% and 88%, respectively, but also inhibited overall fermentation, as suggested by decreased VFA production^17^. In contrast, Dohme et al.^18^ reported no effect of C10 (approximately 600 mg/L) on methanogenesis using a RUSITEC, an in vitro rumen simulation technique. Previous in vitro studies suggested that CH_4_ is reduced by about 5% for each mmol of NT^38,39^. However, in our study, it was only about 3.3% per mmol of NT, indicating an incomplete reduction of NT. Latham et al. (2016)^40^ previously reported that the mitigation potential of NT may be reduced due to its partial metabolization to nitrous oxide either via dissimilatory NT reduction or via incomplete denitrification.

The most efficient binary combination of MCFA in the present study was NT + C10/C12. When expressed as CH_4_ production per VFA, this treatment inhibited CH_4_ production to the greatest extent. Consistent with this, Desbois and Smith^29^ reported that MCFA with 10 or 12 carbons is the most biologically active and that the antimicrobial activity of MCFA decreases with any change in carbon chain length. C12 is one of the most frequently examined MCFAs^19^. In previous studies, C12 has decreased CH_4_ production in the rumen by up to 89% in vitro^18,41^ and by as much as 76% in vivo^42,43^.

The fact that binary combinations of MCFA might be effective anti-methanogenic additives was demonstrated by Soliva et al.^36^ In their study, C12 and C14 exhibited synergistic CH_4_ suppression effects. Their various proportions decreased methanogenesis by 50–96%, and the extent of inhibition increased with increasing amounts of C12 in the mixture. However, in our study, NT + C12/C14 decreased methanogenesis by much less in comparison with the study of Soliva et al.^36^ The lower efficiency of our binary combination may be attributed to the higher concentration used in their study (1000 mg/L vs. 500 mg/L in our study). Another reason could be the C12/C14 ratio. CH_4_ production was not as inhibited in our study as it was in the study of Soliva et al.^36^, where CH_4_ production progressively decreased with an increasing proportion of C12 in the mixture. We assessed a 1:1 ratio, whereas the most effective ratio reported by Soliva et al.^36^ was 2:1 or higher.

In general, the treatments that did not contain C10 or C12 were less effective. Namely, NT with C8, C14, and their binary combination decreased methanogenesis only by 16–24%. In line with this, C14 has been reported to have low efficiency^14,18,20^, and the low potential for CH_4_ inhibition by C8 is in agreement with the findings of Ajisaka et al.^44^.

In general, individual MCFA with NT had little effect on nVFA production or individual VFA proportions. This is in line with the findings of Yanza et al. (2020), who showed that VFA concentrations were not significantly decreased by MCFA in vitro but only in vivo. Furthermore, in our study, sole NT treatment had no effect on nVFA, supporting the results of other in vitro studies with NT^38,45^. The P-value in the analysis of variance was statistically significant (*P* = 0.031); however, Dunnett´s post-hoc analysis failed to identify significant differences between the treatments (*P* > 0.05). Nevertheless, the numerical differences showed the NT + C10 treatment decreased nVFA the most (− 14.2%). Previously, similar doses of C10 (400–600 mg/L) decreased total VFA production by 17–23%^17,18^. This difference could be due to the NT in our treatments, which might have mitigated the inhibitory effects of MCFA on nVFA production, as NT has previously increased VFA concentrations^46,47^. Conversely, treatments C8 and C14 had the lowest effect on rumen fermentation, as indicated by their weak effect on CH_4_ inhibition. In line with the generally low inhibitory effect on CH_4_ production, the effects of the C8 and C14 treatments on nVFA production were negligible.

The molar proportions of the individual VFA were most prominently affected by the NT + C10 treatment. These effects are consistent with the strong anti-methanogenic and antimicrobial activities of C10^29^. The NT + C10 treatment decreased acetate production. Acetate formation, an H_2_ releasing pathway, usually decreases at higher H_2_ concentrations, which occurs when methanogenesis is inhibited^48,49^. High H_2_ concentrations favor H_2_ sinks such as propionate, butyrate, and valerate^48–50^. We did not measure the levels of H_2_, however, this theory was confirmed by an increase in butyrate concentrations, which, as demonstrated in a fermentation balance experiment, may provide 14% of the H_2_ sinks in the rumen^51^. Propionate is a more common H_2_ sink than butyrate^51^; however, its concentration did not increase. This could be because propionate-producing bacteria were inhibited. Indeed, the propionate-producing families *Prevotellaceae*, *Veillonellaceae*, and *Selenomonadaceae*^52,53^ were reduced, but at the expense of *Succinivibrionaceae*, which produce succinate, a precursor to propionate^52^. As a result, another explanation could be that the bacteria producing propionate from succinate were inhibited. Furthermore, the added NT, a favorable H_2_ sink^49^, was available and could have consumed the free H_2_ required for propionate production^54^. Acetate is normally produced by cellulolytic microorganisms along with H_2_^50,55^. The decrease in acetate might be due to C10 inhibiting cellulolytic microorganisms, such as the family *Ruminococcaeae*^56^, which was inhibited in the NT + C10 treatment along with acetate production.

The effects of MCFA on digestibility are type- and dose-dependent^19,31,57^. Higher doses of MCFA typically decrease nutrient digestibility both in vivo and in vitro^19,31,57^. For example, in in vitro continuous culture, C8, C10, and C12 at 5% DM (approximately 600 mg/L) reduced NDF digestion by 2.4, 6.0, and 8.7%, respectively^18^. This reduction may be due to the absorption of fatty acids (FA) on feed particles, limiting the access of enzymes and microbes, and/or FA may be directly absorbed by fiber-degrading microbes (protozoa or cellulolytic bacteria), to which they are toxic^19,24^.

Nevertheless, in the present study, digestibility was not influenced by MCFA, presumably because of their low dosages. The only exception was the NT + C14 treatment (− 6.7%), which had no significant effect on rumen fermentation or nVFA, and the proportion of microorganisms was very similar to that of the control. This result is similar to that of Dohme et al. (2001), who reported the lowest organic matter degradability in C14 (− 7.4%). NT alone (3.65 mM) did not decrease digestibility in our study. Previously, 5 mM NT did not decrease digestibility as well^27^. However, higher doses of NT may be toxic to cellulolytic bacteria and decrease digestibility in vitro^27^.

All treatments, including NT alone, increased pH in our study. This increase may have been due to the addition of NT, which is reduced to ammonia in the rumen. These results are consistent with those of Zhou et al.^26^, who reported an increase in pH at higher NT concentrations. In our study, the NT + C10 treatment increased the pH to a maximum of 6.27, which is in line with the numerically lower nVFA levels in this treatment. Similarly, Dohme et al.^18^ reported the highest pH when supplemented with C10. However, all values of ruminal pH remained within the physiological range (5.5–7.5^54,55^).

Various combinations of MCFA with NT added to ruminal fluid significantly affected the richness and diversity of bacterial populations to different degrees (Table 3, Fig. 1). Currently, treatments containing C10 and/or C12 significantly decreased the alpha diversity indices (Table 3), consistent with their effects on other ruminal parameters in our study and their strong antibacterial activity reported previously^17,18,29^. Burdick et al.^31^ used a mixture of MCFA (C8, C10, and C12) and, contrary to our results, did not report any changes in alpha diversity. This might be due to the low concentrations used in their study, although there was a reported tendency to reduce the bacterial richness. Previous studies on MCFA either did not investigate ruminal microbiota^17,32^ or used less sophisticated (chamber counting method) and insufficiently specific methods^18^.

The dominant bacterial phyla in the current study were *Firmicutes*, *Bacteroidota*, and *Proteobacteria*; this is consistent with the findings of previous studies^47,52,58^. The treatments (particularly those containing C10 and C12) that decreased CH_4_ production also increased the relative abundance of *Proteobacteria* (Fig. 2a). *Proteobacteria* predominantly belonged to the family *Succinivibrionaceae* (Fig. 2c). Hydrogen plays a central role in CH_4_ production^59,60^. The amount of H_2_ in the rumen can be influenced by the abundance of H_2_-producing and H_2_-consuming bacteria associated with CH_4_ emissions^61^. Previously, low-CH_4_-emitting ruminants and tammar wallabies were associated with the H_2_-consuming family *Succinivibrionaceae*^34,35,62^. *Succinivibrionaceae* utilize H_2_ to generate succinate (a precursor to propionate) and, therefore, can reduce CH_4_ emissions^52^. Treatment with NT + C10 increased the relative abundance of *Succinivibrionaceae* the most (70.7%) (Fig. 2c). In contrast, *Succinivibrionaceae* were the least abundant in the NT + C14 treatment (25.2%). NT + C10 and NT + C14 treatments decreased CH_4_ production the most and least, respectively.

Within the phylum *Bacteroidota*, treatments with greater inhibition of methanogenesis decreased the relative abundance of the genus *Prevotella* (Fig. 2d). The genus *Prevotella* utilizes H_2_ and produces propionate^61,63,64^; in a study with Colombian buffalos, a higher abundance of *Prevotella* was associated with lower CH_4_ emissions^65^. However, our findings are not consistent with this, as the genus *Prevotella* was less abundant in treatments resulting in lower CH_4_ production. This lower relative abundance of *Prevotella* could be explained by H_2_ availability in our study. Theoretically, the low H_2_ availability may have been due to the H_2_ being used for the reduction of supplemented NT to ammonia^63,66^. Furthermore, H_2_ could have been utilized by the family *Succinivibrionaceae*, and therefore outcompeted the *Prevotella* genus. This shift in relative abundance from *Prevotella* to *Succinivibrionaceae* has been previously noted^67,68^. Furthermore, the relative abundance of *Prevotella* is decreased by supplementation with NT^69^. However, the majority of the previous studies on NT have reported an increase in the relative abundance of *Prevotella*^12,64,70,71^, because *Prevotella* is associated with nitrate metabolism^71^.

In the phylum *Firmicutes*, treatments with the greatest anti-methanogenic potential (NT + C10 and NT + C10/C12) decreased the relative abundance of the family *Ruminococcaceae* (Fig. 2c). The *Ruminococcaeae* belong to the H_2_-producing bacteria^72^ and are present in higher abundance in high CH_4_-emitting rumens^33,34^. This finding is consistent with our results. However, this family plays a significant role in fiber metabolism, and its reduction may cause a decrease in fiber digestion^73^. Unfortunately, we did not measure fiber digestion, and aDMd was not negatively affected.

A decrease in methanogenesis was also observed in the methanogenic population. The relative abundance of *Archaea,* the sole producers of CH_4_ in the rumen^60^, was decreased by NT + C10 and NT + C10/C12 treatments by 84.2% and 45.7%, respectively (Table 4). Dohme et al.^18^ and Burdick et al.^31^ reported no change in the methanogen population when supplemented with MCFA (C8, C10, and C12). A meta-analysis showed that the *Archaea* population diminished quadratically only under in vitro conditions with increasing doses of MCFA^19^. Furthermore, NT supplementation decreases methanogenesis and consistently reduces the abundance of methanogenic *Archaea*^60,69^. Notably, it has been reported that instead of the overall abundance of methanogens, the community structure of methanogens^60^ and differential gene expression of methanogenesis pathways are the decisive factors in ruminal methanogenesis^74^.

## Conclusion

This in vitro study showed that the effects of NT and MCFA combinations depend on the type of MCFA. The tested treatments have the potential to lower CH_4_ production without negatively affecting ruminal fermentation. The most effective CH_4_ inhibitors were combinations of NT and C10/C12. These combinations also had the greatest impact on the ruminal microbiota, as reflected in the changes in bacterial diversity and shifts in the relative abundances of bacteria and Archaea. The reported results from our 24 h batch incubations should be verified for their long-term effects in vitro and efficiency in vivo.

## Material and methods

### Ethical compliance

Procedures with animals were conducted in accordance with Czech legislation (Act No. 246/1992 Coll., on the protection of animals against cruelty) and applicable European guidelines and regulations (Directive 2010/63/EU, on the protection of animals used for scientific purposes) for experimentation with animals. The experimental protocol was approved by the Animal Ethics Committee of the Institute of Animal Science (Prague, Czech Republic). This study was conducted in accordance with ARRIVE guidelines to ensure an appropriate animal care. The donor cows were housed at the experimental farm of the Institute of Animal Science (Netluky, Prague, Czech Republic).

### Treatments and experimental design

A 24 h batch incubation was used to evaluate the combined effect of NT and MCFA on CH_4_ production and rumen fermentation. The treatments were: control (no NT, no MCFA); NT (nitrate at 3.65 mM), NT + MCFA (nitrate at 3.65 mM + one of four MCFA (C8, C10, C12, and C14) at 500 mg/L), and NT + MCFA/MCFA (nitrate at 3.65 mM + binary combination of MCFA (C8/C10, C8/C12, C8/C14, C10/C12, C10/C14, and C12/C14) at 250 and 250 mg/L). Sodium nitrate was used as the nitrogen source. Three runs were performed over two weeks. In each run, four bottles were assigned to the control (no NT, no MCFA) to obtain a robust average value, three bottles for the NT and blank (no substrate), and two bottles for each of the ten treatments (NT + MCFA and NT + MCFA/MCFA). The average values of the bottles for each treatment in each run were used as experimental replicates.

### Animals, diets, and substrate

Samples of rumen content were manually collected through a rumen cannula (internal diameter, 10 cm) from different sites in the rumen 3 h after morning feeding. Two early lactation Holstein cows (584 ± 20 kg body weight; 32 ± 2 kg of milk/d) were used as donors. Cows were fed a mixed ration consisting (DM basis) of corn silage (337 g/kg), legume-cereal silage (58 g/kg), alfalfa silage (53 g/kg), high-moisture corn silage (50 g/kg), brewer grain (37 g/kg), wheat straw (18 g/kg), liquid supplement (50:50 mixture of molasses and glycerol; 83 g/kg), and concentrate mixture (364 g/kg). The diet was provided twice daily (0400 and 1600) ad libitum.

Rumen content samples were immediately transported to the laboratory in thermal flasks. The interval between sampling and the next sample processing was 20–30 min. In the laboratory, rumen content was strained under continuous CO_2_ flushing through a stainless-steel sieve (250 μm; Retsch, Haan, Germany) to obtain rumen fluid. Rumen fluids from the two donor cows were pooled in equal proportions.

The experimental substrates consisted of (on a DM basis) corn silage (300 g/kg), alfalfa silage (300 g/kg), and barley (400 g/kg). The dried feeds were ground and passed through a 1-mm screen. The chemical composition per kilogram of substrate (DM basis) was as follows: organic matter, 951 g/kg; crude protein, 154 g/kg; ether extract, 25 g/kg; starch, 306 g/kg; neutral detergent fiber (NDF), 354 g/kg; and acid detergent fiber (ADF), 193 g/kg.

### In vitro incubations

The in vitro incubations were conducted over 24 h in 120-mL serum bottles. The dried ground substrate (200 mg) was weighed into sterile CO_2_-flushed serum bottles the day before incubation. The culture fluid (20 mL) was dispensed into each serum bottle using a bottle-top dispenser (Calibrex 530 Salutae, Socorex, Switzerland) under a stream of CO_2_. The culture was prepared by mixing composite rumen fluid with a medium (1:3, v/v) as described previously^75^. The resulting mixture was immediately gassed with CO_2_ at 39 °C for 10 min before being added to the bottles. Sodium nitrate and MCFA were supplied to bottles by adding 200 µL of filter-sterilized distilled water and ethanol stock solutions, respectively, to reach the desired concentration in 20 mL of culture fluid. Equivalent amounts of water and ethanol were added to the control and blank bottles to compensate for the possible effects of solvents on fermentation. The initial headspace gas phase in all incubations was CO_2_. All serum bottles were sealed and placed in a temperature-controlled water bath (SW 22; Julabo, Germany) at 39 °C with a shaking frequency of 90 rpm for 24 h.

### Sampling and chemical analyses

Total volume of gas produced was estimated from the headspace gas pressure using Boyle’s law^76^. Headspace gas pressure was measured using a manometer (Traceable; Fisher Scientific, Pittsburgh, PA, USA) after 24 h of incubation. The headspace gas was then sampled by displacement into a tube (5 mL) prefilled with distilled water by inserting a 23 gauge needle through the stopper of the bottle. The CH_4_ concentration in the headspace gas was measured using gas chromatography^77^.

The concentration of each VFA in the cultures was examined using gas chromatography^77^. VFA concentrations were calculated as the difference between concentrations in the fluid sample after 24 h of incubation and initial concentrations and were therefore reported as nVFA produced. The ammonia-N concentrations in the cultures were determined using the indophenol method^78^. The aDMd was determined gravimetrically by calculating the difference between the weight of the incubated substrate and the dry weight of the fermentation residue, normalized to the residue weight in the blank^77^.

### DNA extraction, polymerase chain reaction (PCR), and amplicon sequence variant (ASV) analysis

DNA (0.5 mL) was extracted from each homogenized sample of the incubation fluid using the repeated bead beating and column purification methods^77^ with modifications. The DNA yield and quality of each sample were quantified using an ND-1000 UV spectrophotometer (NanoDrop Technologies, Witec AG, Littau, Switzerland). The extracted DNA was kept at − 20 °C prior to PCR. The V4 region of the 16S rRNA gene was amplified using the primers 515F (5′-GTGCCAGCMGCCGCGGTAA-3′) and 806R (5′-GGACTACHVGGGTWTCTAAT-3′)^79^. The PCR mixture (20 μL) was prepared using the GoTaq G2 DNA polymerase (Promega, USA); briefly, it consisted of 4 μL 5 × GoTaq buffer, 1.6 μL of magnesium chloride, 1 μL of T4 bacteriophage Gene 32 product, 0.4 μL of each dNTP, 0.1 μL of each primer, 0.1 μL GoTaq Polymerase, 1 μL of template DNA, and 11.1 μL of PCR water.

The PCR reaction was performed with the following steps: initial denaturation at 95 °C for 45 s, annealing at 50 °C for 30 s, elongation at 68 °C for 30 s and a final 5 min extension at 68 °C; the reaction was put to hold at 4 °C. The number of cycles was adjusted separately for each sample to minimize chimeric sequence formation. The resulting PCR products were verified using agarose gel (1%) electrophoresis with staining of the gels with SYBR™ Green I Nucleic Acid Gel Stain (Thermo Fisher, USA). The banding patterns were documented using the Gel Doc XR + System (BioRad, USA). All PCRs were performed in triplicate and pooled.

PCR products were sequenced using the MiniSeq platform (Illumina, USA). The resulting amplicons were analyzed using the DADA2 pipeline (Callahan et al., 2017) and SILVA database (v128)^80^. After normalizing the data to the lowest sample depth (32 000 sequences/sample), relative bacterial abundance was plotted at the phylum (a), class (b), family (c), and genus (d) levels (Fig. 2).

We employed the 'vegan' package in R to visualize and assess community compositional differences (beta diversity) through NMDS (Fig. 3) analysis using the metaMDS function^81^. The ordination patterns were acceptable, as the stress values of the two-dimensional NMDS analysis were below 0.20^82^ (stress = 0.1686036). NMDS stress values are reported after 100 tries and the best solution was repeated 13 times. Next, we utilized ANCOM-BC (version 2.2.2), implemented through the 'ANCOMBC' package^83^, to identify variations in taxa abundance between the groups at the family level (Fig. 4). We applied the Holm-Bonferroni method to adjust for multiple testing and to decrease the likelihood of type I error^84^. Nonsignificant results (*P* > 0.05) were replaced by 0.

### Statistical analysis

The main effect of treatment was analyzed using the PROC MIXED procedure in SAS (SAS Enterprise Guide 6.1, SAS Institute Inc., Cary, NC, USA) according to a randomized complete block design. Runs (n = 3) were the blocking factors. Prior to the statistical analysis, technical replicates (parallel bottles for each treatment) were averaged per run. The model was:

$$Y_{ij} = \mu + T_{i} + R_{j} + e_{ij}$$

where *Y*_*ij*_ is the dependent variable, *µ* is the overall mean, *T*_*i*_ is the fixed effect of the treatment (*i* = 12 levels; control, nitrate, and 10 combinations of nitrate with MCFA), *R*_*j*_ is the random effect of the run (*j* = 1, 2, and 3), and *e*_*ij*_ is the residual error. Treatment means were compared with the control using Dunnett’s adjustment, and differences between each treatment and the control were considered significant at *P* < 0.05.

## Data Availability

The datasets utilized and/or analyzed in the present study can be obtained from the corresponding author upon reasonable request.
